# Prime Editing for Inherited Retinal Diseases

**DOI:** 10.3389/fgeed.2021.775330

**Published:** 2021-11-25

**Authors:** Bruna Lopes da Costa, Sarah R. Levi, Eric Eulau, Yi-Ting Tsai, Peter M. J. Quinn

**Affiliations:** ^1^ Department of Ophthalmology, Columbia University Irving Medical Center, New York, NY, United States; ^2^ Department of Biomedical Engineering, Columbia University, New York, NY, United States; ^3^ College of Arts and Sciences, Syracuse University, New York, NY, United States

**Keywords:** Ophthalmology, prime editing, inherited retinal diseases (IRD), gene editing, retinal degeneration, adeno-associated viral (AAV) vectors, CRISPR/Cas9 systems

## Abstract

Inherited retinal diseases (IRDs) are chronic, hereditary disorders that lead to progressive degeneration of the retina. Disease etiology originates from a genetic mutation—inherited or *de novo*—with a majority of IRDs resulting from point mutations. Given the plethora of IRDs, to date, mutations that cause these dystrophies have been found in approximately 280 genes. However, there is currently only one FDA-approved gene augmentation therapy, Luxturna (voretigene neparvovec-rzyl), available to patients with *RPE65*-mediated retinitis pigmentosa (RP). Although clinical trials for other genes are underway, these techniques typically involve gene augmentation rather than genome surgery. While gene augmentation therapy delivers a healthy copy of DNA to the cells of the retina, genome surgery uses clustered regularly interspaced short palindromic repeats (CRISPR)-based technology to correct a specific genetic mutation within the endogenous genome sequence. A new technique known as prime editing (PE) applies a CRISPR-based technology that possesses the potential to correct all twelve possible transition and transversion mutations as well as small insertions and deletions. EDIT-101, a CRISPR-based therapy that is currently in clinical trials, uses double-strand breaks and nonhomologous end joining to remove the IVS26 mutation in the *CEP290* gene. Preferably, PE does not cause double-strand breaks nor does it require any donor DNA repair template, highlighting its unparalleled efficiency. Instead, PE uses reverse transcriptase and Cas9 nickase to repair mutations in the genome. While this technique is still developing, with several challenges yet to be addressed, it offers promising implications for the future of IRD treatment.

## CRISPR-Based Gene Editing: A Brief Overview

Since the late 1990s, genomic medicine has been at the forefront of gene therapy. While applying genomic medicine to augment gene function has successfully delivered the functional gene to the designated cells, the technique has been limited to correcting loss-of-function alleles and cannot correct gain-of-function mutations (Russell et al., 2017; Tsai et al., 2018; Christie et al., 2020). Additionally, the functional gene is frequently delivered to the target site by viral particles, with larger genes being problematic due to the packaging constraints of the chosen viral vector (e.g., 4.7 kb capacity for AAV and 7–8 kb for lentivirus). The genotoxicity caused by random integration of virus into host genome is also a concern. The transgene can potentially insert into host genome and interfere with DNA transcription/post-transcriptional activity of neighboring genes (David and Doherty, 2017). In contrast, the novel, cutting-edge technique of genome surgery has the potential to directly correct one’s genetic code, addressing the aforementioned barrier faced by gene augmentation for certain mutations. Using clustered regularly interspaced short palindromic repeats (CRISPR) technology, in conjunction with a CRISPR-associated (Cas9) protein and a short sequence of code termed guide RNA (gRNA) designed to target the gene of interest, this technique possessed the machinery to carry out the “cut-and-paste” replacement of the diseased genetic code (Gasiunas et al., 2012; Jinek et al., 2012). Traditionally, this process causes double-strand breaks (DSB) in the DNA. The DSB are repaired by two major pathways in mammalian cells: nonhomologous end joining (NHEJ) and homology-directed repair (HDR). NHEJ is active during the whole cell cycle while HDR is limited to the S/G2 phases. The main difference between these two mechanisms is that NHEJ does not use a template for repair, conversely it randomly corrects the DBS, generating indels (deletions and insertions) at the cut site. On the other hand, HDR uses the sister chromatid as a template for the repair which results in a more precise product (Heyer et al., 2010). A more frequent occurrence than DSB in our body, DNA single-strand breaks (SSB) can arise from spontaneous DNA decay or attack by intracellular metabolites such as reactive oxygen species. There are several SSB repair mechanisms dependent on the source of the break, however, they all follow four main steps: SSB detection, DNA end processing, DNA gap filling and DNA ligation (Caldecott, 2008).

In CRISPR-based technology followed by HDR, the gRNA scans the cell’s nucleus searching for its complementary sequence in the cell’s genome. Upon identification of the corresponding code, the Cas9 precisely interrupts the endogenous DNA causing DSBs and by providing a HDR template the DNA is repaired according to the sequence encoded by this template (Ran et al., 2013). With this technology it is possible to correct many mutations in the DNA which can ultimately restore the synthesis of the healthy mRNA and/or protein. One stark limitation to this process is HDR’s low efficiency due its competition with NHEJ repair mechanism, which is known to be a favorable pathway in mammalian cells (Heyer et al., 2010). Furthermore, given that HDR occurs in the G2 and S phase of the cell cycle, this poses an additional barrier for treatment in nondividing cells, including photoreceptors. Further, the chance random integration of virus during viral delivery of CRISPR/Cas components into host genome can be further boosted by the DSB created by conventional CRISPR machinery (Hanlon et al., 2019). To address the limitations of HDR, a novel method known as base editing was established. Base editing (BE) is a method of genome editing capable of manipulating single-stranded DNA (ssDNA) as opposed to double-stranded DNA (dsDNA). In this way, base editors can forego the process involving DSBs, thereby reducing the rate of indels and making the process more efficient. While this novel technique has eliminated a major complication of CRISPR genome editing, BE was initially designed to install transition mutations in DNA (i.e., A・G to G・A point mutation) (Komor et al., 2016; Gaudelli et al., 2017). Recently, three teams have expanded the capabilities of base editors to install select, one step, transversion mutations in DNA, overcoming some of the previous limitations of this technique (i.e., C・A and C・G point mutations) (Chen et al., 2021a; Kurt et al., 2021; Zhao et al., 2021). However, BE is still not suitable to treat diseases such as sickle cell disease, which is caused by an A to T transversion in the *HBB* gene. Moreover, for precisely installing edits when multiple cytosines or adenines are present within the edit window, or when there is no PAM ideally positioned near the target nucleotide, prime editing (PE) is a valuable alternative approach. However, engineered Cas9s with improvements in PAM flexibility or that are near-PAMless will expand the scope of BE approaches (Kim et al., 2017; Walton et al., 2020).

**FIGURE 1:**
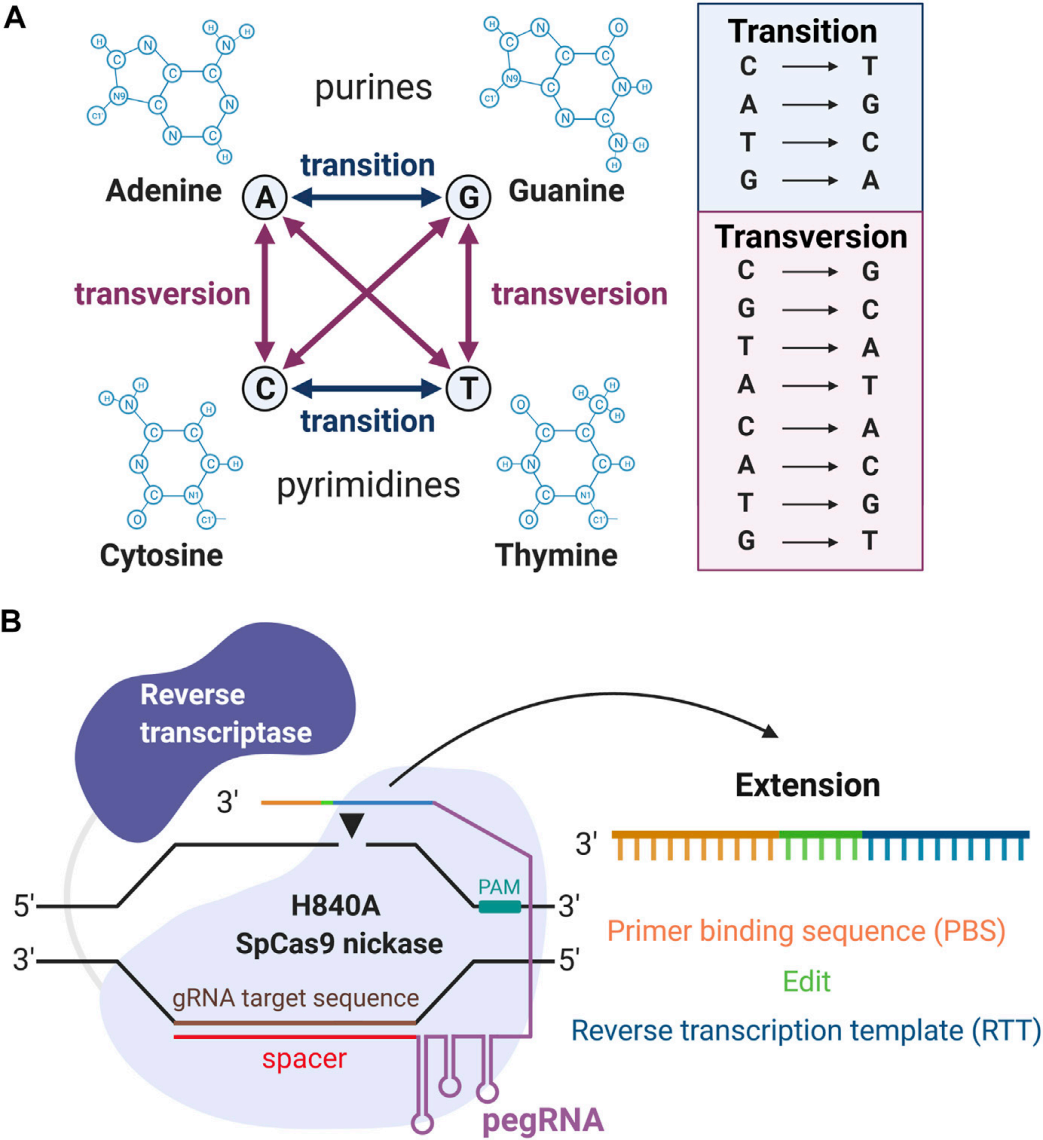
Overview of prime editing (PE). **(A)** Illustrations of all 12 kinds of DNA substitutions. **(B)** The machinery of PE. From the 5′ to the 3′ end, the pegRNA contains the spacer, gRNA scaffold, reverse transcription template (RTT), and primer binding sequence (PBS). “Created with BioRender”.

The “search-and-replace” PE approach, takes gene editing a step further, not only possessing the ability to swap single DNA bases, but also correcting genomic deletions, insertions, and combinations of insertions, deletions, and/or point mutations (Anzalone et al., 2019). Prime editing is the latest gene-editing tool, with both powerful and precise methodology that directly writes new genetic information at the target site. In contrast with the conventional CRISPR-Cas9 approach followed by HDR, PE does not require DSBs nor donor DNA repair templates to precisely edit the human genome. Moreover, PE, contrary to BE, can install all types of transition (interchanges of purines or of pyrimidines), transversion (interchanges of purine for pyrimidine bases, or vice versa) mutations as well as deletions and insertions (Figure 1A) (Anzalone et al., 2019). The unparalleled specificity and efficacy of PE will enable us to optimally repair the specific mutation and ultimately revolutionize the approach to treating inherited retinal diseases (IRDs), including autosomal dominant disorders.

Given the plethora of available techniques for genome editing, PE has proven to be a versatile and powerful approach to CRISPR-based gene repair, sparing any chromosomal DSBs and thus minimizing the adverse effects associated with gene editing (Anzalone et al., 2020). Similarly, by foregoing the use of a single-strand oligonucleotide donor and with PE requiring three hybridization steps between the prime editing guide RNA (pegRNA) and the target site, PE significantly reduces DNA toxicity as well as the possibility of random integration, while lowering the average off-targeting effect to 4.4 times lower than that of CRISPR-mediated HDR (Anzalone et al., 2019). Furthermore, PE has been proven to be more efficient than conventional CRISPR-mediated HDR in gene repair (Anzalone et al., 2019). As we look forward, PE will undoubtedly be the face of gene therapy, and more so, the future of regenerative medicine.

### Mechanism of Prime Editing

**FIGURE 2:**
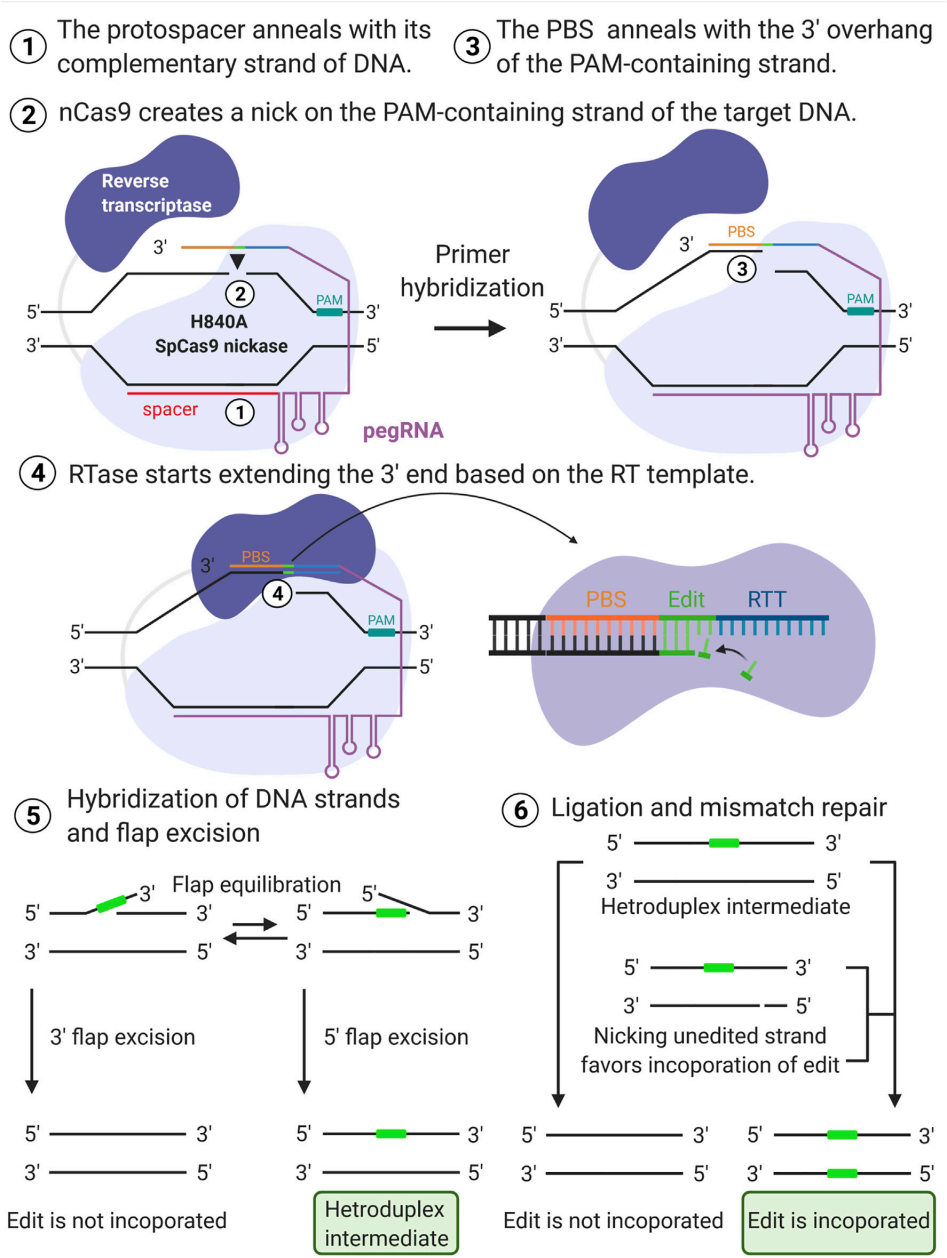
Overview of prime editing mechanism. The spacer (red line) anneals with its complementary strand of the DNA **(1)** directing the H840A SpCas9 nickase to nick the PAM-containing strand (black arrow) of the target DNA **(2)**. The primer binding sequence (PBS) then hybridizes with the nicked DNA **(3)** initiating the elongation of the free 3′ end according to the reverse transcription template (RTT) sequence that carries the intended edit **(4)**. The newly synthesized strand leads to either 3′ or 5′ flap excision. The excision of the 5′ flap is favored, and it leads to the heteroduplex formation **(5)**. The replacement of the original sequence via endogenous DNA mismatch repair mechanism incorporates the desired mutation at the target site **(6)**. “Created with BioRender”.

Prime editing requires two main components: the prime editors that consist of a reverse transcriptase (RT) fused to the H840A SpCas9 nickase, and a pegRNA. The H840A mutation in the conventional SpCas9 inactivates its HNH domain generating a Cas9 nickase, which cleaves only one strand (the PAM containing strand) of the DNA instead of causing DSBs. The pegRNA is designed to extend the 3′ end of the single guide RNA (sgRNA) with a RT template (RTT) and a primer binding sequence (PBS). Thus, from the 5′ to the 3′ ends, the pegRNA contains the spacer, sgRNA scaffold, RTT, and primer binding sequence (PBS) (Figure 1B). An overview of the PE mechanism is illustrated in Figure 2. In summary, the spacer anneals with its complementary sequence, directing the Cas9 nickase to nick the PAM-containing strand of the DNA at a specific locus in the genome. The PBS then hybridizes with the 3′ end of this nicked DNA allowing the RT to carry out the reverse transcription to extend the nicked DNA according to the RTT sequence that carries the intended mutations. This process will create a 3′-flap (the newly synthesized) or a 5′-flap (the original unedited) in this locus. Since the 5′ flap is more susceptible to excision by endonucleases, such as FENI (Liu et al., 2004; Anzalone et al., 2019), the edited strand is more likely to be incorporated in the genome. The excision of the 5′ flap leads to the heteroduplex formation, which is followed by endogenous DNA mismatch repair mechanisms. Finally, the replacement of the original sequence (unedited strand) incorporates the desired mutation at the target site.

### Applications and Current Limitations of Prime Editing

Prime editing is still in its infancy, and further studies are necessary to evaluate its full potential. Herein, we will highlight the current applications and some key limitations of the PE system.

The primary *in vitro* experiments in HEK293T cells showed a high efficiency and flexibly of PE, which was capable of installing different types of editions with high on-targeting rates and low off-targeting edits (Anzalone et al., 2019). Similarly, by using PE system, Schene et al. installed deletions and point mutations in patient-derived intestinal and ductal liver organoids with high editing efficiency (30–50%) and low undesired editing rates (Schene et al., 2020). However, additional studies demonstrated relatively lower rates of editing in hiPSCs, embryos, animals and plants, suggesting that there is large variability in the editing efficiency of this technique (Butt et al., 2020; Hua et al., 2020; Lin et al., 2020; Liu et al., 2020; Sürün et al., 2020). Recently, modifying and incorporating additional nuclear localization signals (NLS) at the N-terminus and C-terminus of the prime editor was found to increase the efficiency of genome editing and should be considered an important parameter in PE design (Liu et al., 2021). In addition, using two pegRNAs *in trans* to install the same modification into the target site, while also overexpressing the pegRNA, has shown to significantly improve the PE efficiency in plants (Jiang et al., 2020; Lin et al., 2021).

The cell type, position in the genome of the target gene, and characteristics of the prime editor—including the number of nucleotides that constitutes the PBS and RTT—all play a critical role in the efficiency of PE (Anzalone et al., 2019). Similarly to the observations made by Anzalone et al. in HEK293 cells, the PBS length showed to critically influence the editing efficiency of PE in hiPSCs (Sürün et al., 2020). In plants, those parameters also showed to be critical. In particular, designing the PBS sequence with a melting temperature of 30°C significantly increases the editing efficiency in rice (Lin et al., 2021). Taken together, these studies demonstrate that optimizing the design of the PE system is crucial and should be performed for each experimental condition. As such, this step can be time and labor consuming. With that in mind, Kim et al., 2021 developed a high-throughput screening method, using a lentivirus library to evaluate the efficiency of the PE2 system (Kim et al., 2021). Although a very interesting approach, the editing efficiency is calculated based on the edition installed on the plasmids, which may not reflect the genomic editing rate that can be complicated by the genomic DNA accessibility. Further studies are necessary to improve the screening method of the PE machinery, including methods that better reflect the *in vivo* efficiency of the selected PE system on the cell type of interest.

Since PE does not induce DSBs, it is expected to have a lower indel rate at the target locus in comparison to NHEJ and HDR. In fact, Anzalone et al. tested this assumption *in vitro* (Anzalone et al., 2019). On the other hand, high levels of unexpected outcomes were installed by the double nicking of PE3 in mouse zygotes (Aida et al., 2020), and a higher frequency of unwanted mutations at target loci were also induced by PE in mice (Liu et al., 2020). On-targeting rates and indel formation are both usually evaluated by next generation sequencing (NGS), followed by bioinformatics analyses, generating individual reads of the region of interest for quantification of on-targeting and indels rates. The parameters used for normalization as well as the number of output reads can significantly affect the calculations of those rates. As such, further *in vivo* studies exploring indel formation at the on-targeting position, including the testing of different bioinformatics parameters, are required.

In terms of off-targeting ratio, researchers frequently identify susceptible off-targeting sites and perform the analysis using techniques such as NGS (Jang et al., 2021), circularization for *in vitro* reporting of cleavage effects by sequencing (CIRCLE-seq) (Levy et al., 2020; Suh et al., 2021), and nickase-based Digenome sequencing (nDigenome seq) (Kim et al., 2020). With that, the off-targeting rates can be often underestimated since only few predicted off-targeting sites are studied. Therefore, it is still necessary to evaluate the *in vivo* PE off-targeting frequency in a genome-wide respect.

Until now, only a few studies exploring PE *in vivo* have been conducted with its efficiency notably lower than it is *in vitro* (Liu et al., 2020; Liu et al., 2021; Gao et al., 2021). Due to the large size of PE machinery, it’s *in vivo* delivery to the target site can be a limitation that significantly impacts the editing efficiency of this technique. Recently, Liu et al. tested a dual adeno associated virus (AAV)-mediated delivery of a split-intein prime editor and showed its applicability for *in vivo* gene editing in the mouse liver (Liu et al., 2021). However, this strategy still requires the use of dual-AAV vectors, which causes concern regarding the expression of undesired truncated products and low efficiency. By using dual vectors system, both vectors must reach the target site at the same time in order to guarantee a successful outcome.

Prime editing is a cutting-edge technique that holds great promise to the advance of genome engineering. Future research should focus on applying this technique in a plethora of human disease-relevant cell types, organoids and animal models to support the clinical potential of PE in treating human genetic diseases.

### DSB Independent Technology for Inherited Retinal Dystrophies

CRISPR/Cas systems are a promising avenue for the treatment of IRDs. Specifically, given the eye’s unique immune-privileged nature, this organ presents an opportunity for CRISPR/Cas-mediated treatment of IRDs with limited systemic effects (Benhar et al., 2012). The significant progress being made in this field is exemplified by the ongoing phase I/II clinical trial for EDIT-101 (AGN-151587), a treatment for Leber congenital amaurosis (LCA) type 10 (Accessed on September 2021: https://clinicaltrials.gov/ct2/show/NCT03872479). EDIT-101 removes the aberrant splice donor created by the *CEP290* IVS26 c.2991 + 1655 A > G mutation in the *CEP290* gene through AAV5-mediated delivery of dual gRNAs in conjunction with the Cas9 ortholog from *Staphylococcus aureus* (Maeder et al., 2019). However, the development of DSB-independent CRISPR/Cas systems—such as PE and BE—that significantly reduce off-targeting effects as well as the introduction of indels at the editing site, will be a significant future step for IRD therapeutics. Here, we provide a brief update on DSB-independent retinal therapeutics. For a detailed overview, Gallego et al*.* discuss CRISPR/Cas-based gene editing approaches for IRDs (Gallego et al., 2020).

A recent retrospective analysis conducted by Fry et al. examined the prevalence of single nucleotide pathogenic variants with the potential to be corrected by BE. Specifically, this study identified 6 autosomal recessively inherited genes associated with IRDs; namely, *ABCA4, CEP290, CDH23, EYS, MYO7A*, and *USH2A* whose long coding sequence prevents them from being corrected with a classical single AAV-mediated gene augmentation strategy but have variants amenable for correction by BE (Fry et al., 2021). Prime editing, due to its potential to rectify 89% of pathogenic genetic variants, would further expand the therapeutic editing possibilities for the aforementioned genes (Anzalone et al., 2019). Base editing has been applied for the treatment of retinal degeneration 12 (*rd12*) mice, a representative model of humans with *RPE65* mutations (Suh et al., 2021). Here, Suh et al. corrected the homozygous C > T nonsense mutation in exon3 of the *Rpe65* gene, finding as high as 29% editing efficiency, through lentiviral-mediated delivery of an adenine base editor (ABE) and sgRNA. Importantly, they found minimal indels or off-target mutations and the mice had restored RPE65 expression. They additionally found the recovery of retinoid isomerase activity after BE, with a substantial increase in 11-*cis*-retinal in treated eyes leading to functional visual recovery as measured by electroretinography (ERG), optomotor responses (OMRs) and visually evoked potentials (VEPs) (Suh et al., 2021).

Split-intein ABEs and cytosine base editors (CBEs) have also been developed and showed favorable transduction efficiency when delivered by dual AAVs (either the evolved PHP.B or Anc80 AAV capsids) to the retina (Levy et al., 2020). Rhodopsin-Cre mice were crossed with Ai9 mice to generate mice that expressed tdTomato only in rod cells. These mice were injected at 2-weeks-old with the AAV-ABE and AAV-CBE constructs to target the *Dnmt1* locus, in addition co-injection with their corresponding reporter constructs PHP.B-CBh-GFP–KASH or Anc80-CBh-GFP–KASH with the nuclear membrane-localized Klarsicht/ANC-1/Syne-1 (KASH) homology driven by chicken-beta hybrid (CBh) promoters. At 3-weeks post-injection, efficiency was determined by assessing editing in sorted cells. The authors found 48 ± 5.9% C•G-to-T•A editing with PHP.B-CBE and 37 ± 22% A•T-to-G•C editing with Anc80-ABE in GFP^+^/tdTomato^+^ transduced rod photoreceptors. However, while ABE delivery led to the generation of minimal indels in retinal cells, CBE delivery to retinal cells generated substantial indels of up to 34% (Levy et al., 2020). Interestingly, there was minimal overlap between base-edited and indel-containing alleles. One possibility proposed by the authors is that CBE-mediated indels may occur at a higher rate in retinal cells due to mutual exclusivity between uracil excision pathways and those pathways required for CBE-mediated editing outcomes (Levy et al., 2020). Recently, AAV8-mediated delivery of split-PEs has also been shown to successful edit the *Dnmt1* locus in the mouse retina using a CMV promoter (Zhi et al., 2021). In this study, Zhi *et al.* sub-retinally co-injected their split-intein PE along with an AAV8-CMV-GFP reporter at 6 weeks of age and found expression limited to photoreceptors and retinal pigment epithelium (RPE). At 6 weeks post-injection, genomic DNA of mouse retina was collected (no cell sorting). This revealed an average editing efficiency of 1.71 ± 1.35% and average indels of 0.17 ± 0.01% in *Dnmt1* locus (Zhi et al., 2021). Similarly, a preprint by Jang *et al.* showed an editing efficiency of 1.87% in the *Atp7b* locus in transduced mouse retina (no cell sorting) and no detectable indels were found. Jang and others used a *trans*-splicing AAV8 vector, which allows the expression of a single transcript encoded by two independent vectors coadministered to the same tissue to deliver the PE to the retina via intravitreal injection, in conjunction with an additional AAV8 construct to deliver the pegRNA and sgRNA (Jang et al., 2021).

Lastly, the *rd10* mouse model mimics autosomal recessive retinitis pigmentosa (RP) and is caused by the *Pde6b* ^*rd10*^ c.1678C > T (p.Arg560Cys) mutation. To date, neither the PE nor the BE approach has been used to correct the *rd10* model. However, Vagni and others illustrated the amenability of the *rd10* model to *in vivo* treatment using CRISPR/Cas9-mediated HDR (Vagni et al., 2019). Their findings revealed a higher visual acuity compared to the untreated eye 3 months post gene editing (Vagni et al., 2019). Recently, our laboratory demonstrated the applicability of PE for the successful installation and correction of the *Pde6b* ^*rd10*^ c.1678C > T mutation *in vitro* using the Neuro-2a (N2a) mouse neuroblastoma cell line (Tsai et al., 2021). We hope this proof-of-concept work will pave the way for future *in vivo* studies on the applicability of PE for IRDs.

Taken together, this information continues to point towards the advancement of CRISPR genome editing techniques and their application in the field of ophthalmology. As we continue down this path, BE and PE approaches will ultimately be at the forefront of ophthalmic gene therapy.

## Conclusion

DSB- and cell-cycle- independent retinal therapeutics hold immense implications for the treatment of IRDs in the years to come. Although still in the development stage, DSB-independent therapeutics may soon become the most broadly used treatment for mutations leading to IRDs. Prime editing expands on the capabilities of BE by enabling correction of all twelve possible transition and transversion mutations along with small insertions and deletions. Therefore, PE may be the most flexible, precise, and least risky option for altering point mutations to date. Safety and high editing efficiencies are the parameters that scientists aim to achieve, and in this sense optimizations of PE will drive the future research in the field. To illustrate this trend, David Liu’s group published a paper where they incorporated structured RNA motifs to the 3′ terminus of pegRNAs as a strategy to decrease its degradation by exonucleases and therefore increase editing efficiency (Nelson et al., 2021). Further, Liu group recently found that manipulating mismatch repair by temporarily inhibiting a component of mismatch repair significantly increased editing efficiency and produced fewer indels. Interestingly, in the same study Chen et al. found that installation of silent mutation increased PE efficiency by evading mistmatch repair mechanisms (Chen et al., 2021b). Therapeutic editing is a rapidly evolving field, and as it continues to make strides forward, user-friendly PE design tools are automating, simplifying, and decreasing the barriers to utilize this technology (Bhagwat et al., 2020; Chow et al., 2021; Hsu et al., 2021; Siegner et al., 2021; Standage-Beier et al., 2021). Further, prime editors that possess increased PAM flexibility have been generated, broadening the scope of this methodology (Kweon et al., 2021). However, efficient delivery of PE machinery must be further optimized and, more significantly, there is insufficient insight into the long-term safety profile of PE. Research to better understand the cellular repair mechanisms triggered by PE, the off-targeting effects in terms of whole genome and possible delivery vectors, including non-viral systems, such as liposomes, would significantly contribute to the PE field. The testing and optimization of PE using patient-derived induced pluripotent stem cells and subsequently derived ophthalmic organoids is an exciting perspective for the development and future of this technology.
